# Role of fibroblast growth factor-23 as an early marker of metabolic bone disease of prematurity

**DOI:** 10.1186/s12887-024-04897-7

**Published:** 2024-06-29

**Authors:** Sandra Llorente-Pelayo, Pablo Docio, Silvia Arriola, Bernardo A. Lavín-Gómez, María T. García-Unzueta, María Ángeles Ballesteros, María J. Cabero-Pérez, Domingo González-Lamuño

**Affiliations:** 1 Pediatric Department, University Hospital Marqués de Valdecilla—Research Institute Valdecilla (IDIVAL), Santander, 39008 Spain; 2 Neonatology Unit, Pediatric Department, University Hospital Marqués de Valdecilla—Research Institute Valdecilla (IDIVAL), Santander, 39008 Spain; 3 Biochemical Department, University Hospital Marqués de Valdecilla—Research Institute Valdecilla (IDIVAL), Santander, 39008 Spain; 4 Department of Critical Care Medicine, Hospital Marqués de Valdecilla-IDIVAL, Avda Valdecilla s/n, Santander, 39008 Spain; 5 Departamento de Ciencias Médicas y Quirúrgicas, University of Cantabria, Santander, 39005 Spain

**Keywords:** Metabolic bone disease of prematurity, Prematurity, Phosphorous, Fortification

## Abstract

**Purpose:**

Metabolic bone disease of prematurity (MBDP) remains a significant cause of morbidity in extremely premature newborns. In high-risk patients, suspected diagnosis and subsequent treatment modifications, with limitations in terms of sensitivity and specificity, rely on low phosphorus levels and/or high levels of alkaline phosphatase (ALP). We investigated the potential of fibroblast growth factor-23 (FGF23) as an early marker for MBDP when measured at 3–4 weeks of life in at-risk patients.

**Methods:**

A single-center prospective observational non-interventional study including preterm newborns of both sexes, with a gestational age of less than 32 weeks and/or a birth weight of less than 1500 g. In the standard biochemical screening for MBDP performed between 3 and 4 weeks of life within a nutritional profile, the determination of FGF23 was included along with other clinical and metabolic studies. The study was conducted at Marqués de Valdecilla University Hospital in Santander, Spain, from April 2020 to March 2021. Participants provided informed consent. Biochemical analyses were conducted using various platforms, and follow-up evaluations were performed at the discretion of neonatologists. Patients at high risk for MBDP received modifications in treatment accordingly. The sample was descriptively analyzed, presenting measures of central tendency and dispersion for continuous variables, and absolute numbers/percentages for categorical ones. Tests used included t-tests, Mann‒Whitney U tests, chi-square tests, logistic regressions, Pearson correlation, and ROC curve analysis (IBM SPSS Statistics version 19). Significance level: *P* < 0.05.

**Results:**

In the study involving 25 at-risk premature newborns, it was found that 20% (*n* = 5) were diagnosed with MBDP. Three of these patients (60%) were identified as high-risk based on standard biochemical evaluation at 3–4 weeks of age, while the other two patients (40%) were diagnosed in subsequent weeks. However, in all 5 patients, measurement of FGF23 levels would allow for early identification and optimization of treatment before other markers become altered. Low levels of FGF23 at 3–4 weeks, even with normal phosphorus and ALP levels, indicate the need for modifications in nutritional supplementation.

**Conclusions:**

MBDP remains a significant concern in extremely premature newborns. Current diagnostic methods rely on limited biochemical markers. Early detection of low FGF23 levels enables timely interventions, potentially averting demineralization.

**Supplementary Information:**

The online version contains supplementary material available at 10.1186/s12887-024-04897-7.

## Key points

***Question***: Is fibroblast growth factor-23 (FGF23) measured at 3–4 weeks of life a reliable early marker for metabolic bone disease of prematurity (MBDP) in at-risk preterm newborns?

***Findings***: This study was a single-center prospective observational noninterventional study conducted in a neonatal unit in Spain. The primary outcome was to investigate FGF23 as a potential early marker for MBDP. The study found that low levels of FGF23 at 3–4 weeks of life, even with normal phosphorus and alkaline phosphatase levels, indicated the need for modifications in nutritional supplementation. FGF23 levels at this time point were found to predict subsequent hypophosphatemic status, making it the most sensitive early marker for MBDP in at-risk preterm newborns.

***Meaning***: Early detection of low FGF23 levels at 3–4 weeks of life can help identify at-risk preterm newborns with MBDP and prompt timely treatment modifications, potentially preventing demineralization and improving outcomes in this vulnerable population.

**Supplementary Information:**

The online version contains supplementary material available at 10.1186/s12887-024-04897-7.

## Introduction

Metabolic bone disease of prematurity (MBDP), also known as osteopenia or rickets of prematurity, is a common condition characterized by mineralization disorders in premature newborns [1, 2]. Fetal bone mineralization primarily occurs during the third trimester, with most calcium and phosphate accumulation taking place during this period [3]. As a result, MBDP is frequently observed in infants born before completing 32 weeks of gestation [4], affecting a significant proportion of very low birth weight (VLBW) and extremely low birth weight (ELBW) infants [5–7].

In addition to inadequate mineral supply, other contributing factors to MBDP include chronic diseases, immature gastrointestinal tract, hormonal imbalance, and the use of medications such as diuretics and steroids [8–10]. The optimal calcium and phosphorus requirements for preterm infants are not well defined, and there is a lack of consensus in recommendations [2, 8, 11–15]. Challenges in achieving optimal mineral levels include the decreased stability of parenteral nutrition with mineral supplements, delayed initiation of enteral feeding, and irregular absorption and bioavailability of oral mineral supplements [16–18].

Demineralization in MBDP occurs progressively in the first weeks after birth, but infants often show few symptoms or signs until the later stages of demineralization, typically between 6 and 16 weeks after birth [6, 19]. Initial indicators are usually biochemical, with elevated serum alkaline phosphatase (ALP) followed by radiological signs such as osteopenia and pathological fractures. MBDP can lead to restricted growth velocity, fractures, pain, respiratory distress, and impaired pulmonary function. If left untreated, severe rickets may develop [2, 6, 19, 20].

The assessment of long-term consequences of MBDP is challenging due to difficulties in defining the diagnosis and confounding factors, but evidence suggests an association between MBDP and poor childhood growth [19, 21, 22]. Early identification of the risk of MBDP is crucial to initiate timely therapeutic interventions [2], as optimal nutritional supplementation and other measures have been shown to prevent MBDP [8, 11–15].

In clinical practice, specific radiological techniques for quantifying bone mineral content, such as X-ray absorptiometry (DXA) or quantitative ultrasound (QUS), are typically unavailable [23–26], and the suspected diagnosis and subsequent therapeutic interventions rely on biochemical markers, which can be assessed earlier [27]. However, none of the bone metabolism markers can be considered specific to MBDP. Therefore, there is a lack of universally accepted management protocols, and variations exist between countries and centers.

Phosphorus levels and ALP are commonly used markers for MBDP screening, along with parathyroid hormone (PTH) levels [8]. Low phosphorus and elevated ALP levels have been associated with MBDP. However, these markers are not specific to the condition. The combination of high ALP levels and low phosphorus levels has shown moderate sensitivity and specificity for the diagnosis of MBDP [28–32].

Despite significant advances in neonatal care, MBDP remains a frequent cause of morbidity in extremely premature newborns, and there is no universal consensus on the screening, diagnosis, and management of this condition. Therefore, there is a need to explore the usefulness of early mineral metabolism markers in the management of MBDP.

Fibroblast growth factor-23 (FGF23) plays a crucial role in mineral metabolism, particularly in regulating phosphate and vitamin D homeostasis. It is primarily produced by osteocytes in bone tissue and acts on various target organs, including the kidneys and parathyroid glands [33, 34].

One of the main functions of FGF23 is to regulate phosphate levels in the blood. It does so by inhibiting phosphate reabsorption in the kidneys and suppressing the production of active vitamin D (calcitriol) in the kidneys. By reducing phosphate absorption and vitamin D synthesis, FGF23 helps maintain phosphate balance in the body [35, 36].

The reduced active vitamin D in turn impairs calcium absorption in the intestines and contributes to hypocalcemia [37].

FGF23 is particularly relevant in the context of metabolic bone diseases, such as osteoporosis and rickets, because dysregulation of FGF23 levels can disrupt mineral metabolism and bone health [38, 39]. In conditions where FGF23 is excessively produced or dysregulated, such as X-linked hypophosphatemia or tumor-induced osteomalacia, patients often present with abnormalities in bone mineralization and skeletal deformities [40, 41].

Furthermore, FGF23 has emerged as a potential biomarker for assessing metabolic bone disease risk in premature infants. By understanding the mechanisms of FGF23 and its role in mineral metabolism, clinicians can utilize FGF23 levels as a biomarker to monitor bone health and identify individuals at risk of metabolic bone diseases, particularly in vulnerable populations such as premature infants. Additionally, further research into the regulation of FGF23 and its interactions with other hormones involved in mineral homeostasis may lead to novel therapeutic strategies for managing metabolic bone diseases [42, 43].

This study aims to investigate the potential of FGF23 levels at 3–4 weeks of life as an early and sensitive marker of subclinical hypophosphatemia and high risk for MBDP. By evaluating FGF23 levels in a small cohort of premature infants, the study aims to contribute to the understanding of early markers and improve the management of MBDP in this population.

## Patients and methods

The present study is a single-center prospective observational noninterventional study including preterm newborns of both sexes, with a gestational age (GA) of less than 32 weeks and/or a birth weight (BW) of less than 1500 g. The study was conducted in the neonatal unit of the Marqués de Valdecilla University Hospital in Santander, Spain, between April 2020 and March 2021. Patients who died before one month of life or did not provide informed consent were excluded. Informed consent was obtained from all participants (parents or guardians) included in the study, in accordance with the criteria of the Ley de Investigación Biomédica 14/2007. The study was conducted in accordance with the Declaration of Helsinki and approved by the IRB (Comité de Investigación con Medicamentos de Cantabria; reference 2019.294) and the Ethical Committee of the authors’ hospital (CEIC-C: Comité Ético de Investigación Clínica de Cantabria, project number CSI20/09).

Clinical, biochemical, and radiological variables were collected from birth to discharge from the neonatal unit. The clinical variables included sex, gestational age, biometrics at birth (including intergrowth 21th percentile), weight and height evolution during hospitalization, day of first mobilization for Kangaroo mother care, nutrient intake (amount, type of nutrition, including specific calcium and phosphorus intakes), and all comorbidities. All nutritional intakes and medications received at the time of biochemical extraction were specifically collected.

Single serum, plasma, and isolated urine samples were collected between weeks 3 and 4 of life, coinciding with the usual extraction of the nutritional profile and the standard biochemical screening of MBDP. The samples were collected as specified for the different assays and centrifuged, and aliquots for this specific study were stored at -80 °C until analysis in the Biochemistry Laboratory of the Marqués de Valdecilla University Hospital.

Blood metabolic variables, including urea, creatinine (Cr), total calcium (Ca), phosphorus (P), magnesium (Mg), alkaline phosphatase (ALP), and parathyroid hormone (PTH), among others, were analyzed using the Atellica CH&IM platform (Siemens Healthcare Diagnostics, Malvern, PA, USA). Cystatin-C (CysC) was analyzed using the BNII-Nephelometer System (Siemens Healthcare Diagnostics, USA). Levels of 25-hydroxycholecalciferol (25-OH-Vit D or calcidiol) were measured using an automated competitive chemiluminescence assay (Liaison XL, DiaSorin Inc, Stillwater, MN, USA), and levels of 1,25-dihydroxycholecalciferol (1-25-2OH-vit D or calcitriol), bone alkaline phosphatase (B-ALP), and plasma fibroblast growth factor-23 (FGF23) were measured using enzyme-linked immunosorbent assay (ELISA) kits. The ELISA kits used were as follows: 1,25(OH)2 Vitamin D ELISA (DiaSource Immunoassays, Belgium), MicroVue Bone Alkaline Phosphatase EIA (Quidel-Palex Medical, Barcelona, Spain), and Human FGF23 (Intact) ELISA from Immunotopics International (San Clemente, CA 92,673). The FGF23 ELISA kit used has the following features: 3.5% intra-assay and 6.3% interassay reproducibility, it does not cross-react with the N-terminus (25–179) or C-terminus (180–251 fragments), and the limit of quantification is 5 ng/L.

Urinary variables and their ratios by creatinine (uCr), including calcium (uCa/uCr), phosphorus (uP/uCr), gamma-glutamyl transferase (uGGT/uCr), and cystatin-C (uCysC/uCr), were analyzed at the same time using the Atellica platform and BNII-Nephelometer (Siemens Healthcare).

Subsequent biochemical controls were performed at the discretion of the responsible neonatologist for each patient. Patients with serum phosphorus levels lower than 4 mg/dL (or 1.29 mMol/L) and/or serum ALP levels above 750 IU/L at any time during the follow-up were classified as high risk for MBDP, and subsequent modifications in treatment were performed at the discretion of the responsible neonatologist. Individual instrumental studies were used according to the clinical situation of each patient. No specific radiological studies, DXA, or QUS were established in our center during the follow-up.

### Data analysis

A descriptive analysis of the sample was performed. The results are presented as measures of central tendency and dispersion for continuous variables and as absolute numbers and percentages for categorical variables. The main outcome studied was MBDP.

Student’s t test, Mann‒Whitney U test, or median tests were used for continuous variables, depending on the distribution of the sample. The Kolmogorov‒Smirnov test was used to identify variables with a normal distribution. Student’s t test, Mann‒Whitney U test, or median tests were used for continuous variables, depending on the distribution of the sample. The Kolmogorov‒-Smirnov test was used to identify variables with a normal distribution. The chi-square test and Fisher’s exact test were used for comparisons of categorical variables.

Simple logistic regression and multivariate logistic regression analyses were used, and each independent variable was analyzed together with the dependent variable MBDP. Pearson correlation analysis was performed to explore the relationship between serum biochemical markers.

To evaluate the potential biomarkers responsible for predicting metabolic bone disease of prematurity, the receiver operating characteristic (ROC) curve was used. The cutoff point for each potential biomarker was also calculated from the sensitivity vs. 1-specificity plot. The accepted level of significance was set at *P* < 0.05. Statistical analysis was performed using IBM SPSS Statistics version 19 software.

## Results

Between April 2020 and March 2021, a study was conducted on 25 preterm patients born at a neonatal unit. Out of the initial 29 eligible patients, four died. Among the included patients, 60% were female, and there were six pairs of twins. The median gestational age was 30 weeks, ranging from 24 to 34 weeks, with a median birth weight of 1180 g, ranging from 645 to 2030 g. 20% of the patients were classified as having a low weight for gestational age. During the follow-up, 20% of the group was diagnosed with MBDP, with one patient presenting with pathological fractures. The diagnosis of MBDP in our study was established through a multifaceted approach encompassing clinical evaluation, radiographic assessment, and laboratory analysis. The main characteristics of the study population are summarized in Table 1.

**Table 1:** Characteristics of the study population

Gestational age	Median 30 weeks (IQR 26.6–31.2)
Anthropometry at birth	
- Birth Weight	Median 1180g (IQR 900–1445)
- Percentile Birth Weight	Median 54.12 (IQR 21.63–70.98)
- Birth size	Median 38 cm (IQR 35.5–40.5)
- Percentile Birth Size	Median 38 (IQR 35.5–40.5)
- Birth Cephalic Perimeter	Median 27 cm (IQR 24-28.75)
- Percentile Cephalic Perimeter	Median 37.5 (IQR 11.72-69)
First mobilization (Kangaroo mother care)	Median 5 days (IQR 2.5-13-5)
Medical conditions	
- MBDP	5 (20%)
- Respiratory conditions	
Apnea of prematurity	20 (80%)
Hyaline membrane disease	17 (68%)
Bronchopulmonary dysplasia	8 (32%)
- Cardiac conditions:	
Patent ductus arteriosus	9 (36%)
Other cardiopathies	2 (8%)
- Endocrine and metabolic disorders:	
Early neonatal hypoglycemia	14 (56%)
Hypothyroidism	6 (24%)
- Hematological disorders:	
Anemia	10 (40%)
Other disorders	4 (16%)
- Neurological disorders:	
Intraventricular hemorrhage	5 (20%)
Seizures	2 (8%)
- Neonatal jaundice	13 (52%)
- Neonatal cholestasis	2 (8%)
- Necrotizing enterocolitis	2 (8%)
- Infectious diseases:	
Chorioamnionitis	10 (40%)
Sepsis	5 (20%)
Pneumonia	4 (16%)
Meningitis	1 (4%)
Peritonitis	1 (4%)
Viral infections	5 (20%)
- Surgical procedures:	
Intestinal resection	1 (4%)
Ductus closure	1 (4%)

Note: IQR denotes the interquartile range, which represents the range between the 25th and 75th percentiles

All patients received parenteral nutrition (PN) at birth, with an average duration of 9 days (IQR 5.5–12.5). 24% of patients received PN for more than 2 weeks. Enteral nutrition (EN) was initiated during the first day of life for 80% of the patients. However, during the first month, 16% of patients remained on exclusive parenteral nutrition for more than 3 days (from 4 to 12 days). The median duration of EN using tube feeding was 42 days until full volume oral nutrition was achieved (IQR 21-74.5). The median maximal weight loss was 9.9%, and the median weight gain in the first month was 510 g (IQR 275–680), calculating a weight gain of 29.28 g/day (IQR 16.78–36.06) by first month of life. At the time of the biochemical analysis, 96% of patients were on exclusive enteral nutrition, 24% with preterm formula, 72% with fortified human milk and 4% with a mix of preterm formula and fortified human milk. Fortification of human milk was started at a mean of 9 days. All patients received phosupplementation, with a mean dose of 600 IU/day for vitamin D. The average caloric intake at 3–4 weeks of life was 130.8 Kcal/kg/day (IQR 124.1-142.2), with mean calcium and phosphorus intakes of 146 mg/kg/day (IQR 134.3-183.3) and 83.6 mg/kg/day (IQR 72.4-100.4), respectively. All the patients, except for one, were receiving appropriate supplies according to ESPGHAN recommendations. The mentioned patient (patient #13) was receiving a mix of enteral and parenteral nutrition with adequate supplies of energy and phosphorus but low calcium intakes, with a decreased calcium-phosphorus ratio. Nutritional parameters can be found in appendix 1.

In the biochemical evaluation, the median phosphorus level during weeks 3–4 of life was 6.9 mg/dL (IQR 6.2–7.3), with median calcium levels of 9.6 mg/dL (IQR 9.450-10) and a median magnesium level of 1.9 mg/dL (IQR 1.5-2). The median PTH level was 58 pg/mL (IQR 32.25–75.25), with three patients having levels above 88 pg/mL. The median calcidiol level was 27 ng/mL (IQR 20.5–33), and the median calcitriol level was 102 pg/mL (IQR 81–141). At one month of life, the median ALP value was 333 IU/L (IQR 286.5–485), and the median B-ALP level was 139 IU/L (IQR 106–186). Both phosphorus and ALP values ​​were very normal for patients at risk for MBDP.

The median uCa/uCr ratio was 0.6 mg/mg (IQR 0.31–0.79), and the uP/uCr ratio was 1.78 mg/mg (IQR 0.51–2.59), with a TRP of 92% (IQR 86.77–97.69). The median uGGT/uCr ratio was 1.77 mg/mg (132,5-366,67).

FGF23 levels ranged from 5 to 101.20 ng/L, with a median of 45.9 ng/L (IQR 13.02–76.77 ng/L).

**Fig. 1:**
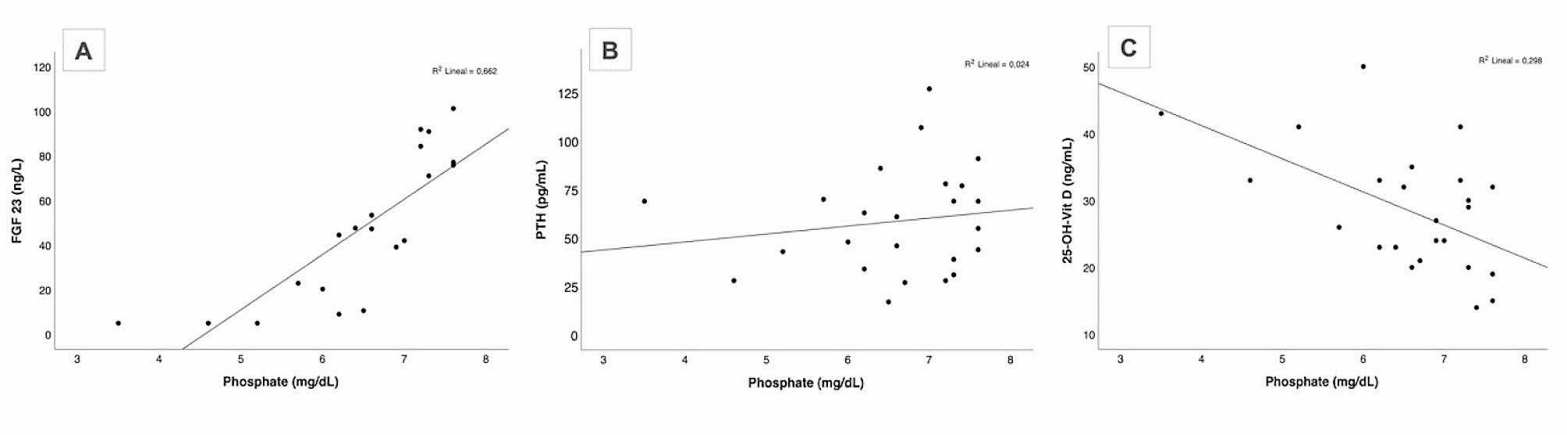
Correlation plots: **A**- Correlation between FGF23 and phosphate. **B**- Correlation between PTH and phosphate. **C**- Correlation between 25-OH-VitD and phosphate FGF23: Fibroblast growth factor-23; PTH: parathormone

A strong correlation was observed between plasma phosphorus and FGF23 levels in the preterm samples (correlation coefficient of 0.824, *p* < 0.001) (Fig. 1). No significant correlation was found between phosphorus and PTH levels, but there was a slight association between phosphorus and a high uCa/uCr ratio (-0.44, p 0.028). A high uCa/uCr ratio was also related to low PTH levels (-0.657, *p* < 0.001). We did not find a significant association between FGF23 ​​and the uP/uCr ratio or TRP. There was also no correlation between tubular urinary function (uCystatinC/uCr and uGGT/uCr) and gestational age or corrected age.

### Clinical factors associated with MBDP

Several clinical factors were found to be associated with MBDP (Table 2). Lower gestational age and birth weight were expectedly linked to MBDP, with MBDP patients having a median gestational age of 26.4 weeks compared to 30.85 weeks in non-MBDP patients. Similarly, the median birth weight was lower in MBDP patients (900 g) than in non-MBDP patients (1275 g) (*p* < 0.021). However, no significant association was observed between MBDP and birth weight percentile.

**Table 2:** Clinical factors associated with MBDP

	All (25)	MBDP (5)	No MBDP (20)	*p*
Gestational age	30 w (IQR 26.6–31.2)	26.4 w (IQR 25.6-27.64)	30.85 w (IQR 27.89–31.54)	**0.021** ^**a**^
Birth weight	1180 g (IQR 900–1445)	900 g (IQR 765–950)	1275 g (IQR 987.5-1568.75)	**0.021** ^**a**^
Other comorbidities:				
-BPD	8 (32%)	5 (100%)	3 (15%)	**0.001** ^**b**^
-Pneumonia	4 (16%)	4 (80%)	0	**< 0.001** ^**b**^
-Hypothyroidism	6 (24%)	4 (80%)	2 (10%)	**0.005** ^**b**^
-Anemia	10 (40%)	5 (100%)	5 (25%)	**0.005** ^**b**^
-Patent ductus arteriosus	9 (32%)	4 (80%)	5 (25%)	**0.04** ^**b**^
-Seizures	2 (8%)	2 (40%)	0	**0.033** ^**b**^
First day mobilization KMC	5 d (IQR 2.5–13.5)	34 d (IQR 8–52)	4 (IQR 2-11.2)	**0.008** ^**a**^
Weigh gain during first month	29.3 g/d (IQR 17.9–35.7)	17.9 g/d (IQR 10.7–18.6)	30.4 g/d (22.9–39.6)	**0.035** ^**a**^
Days on exclusive PN during first month	0 d (IQR 0–2)	4 d (IQR 2–7)	0 d (IQR 0–0,5)	**0.001** ^**a**^
Days on PN during first month	9 d (IQR 6–11)	15 d (IQR 11–19)	8 d (IQR 5–10)	**0,007** ^**a**^
Days on NGT feeding	42 d (IQR 21–71)	90 d (IQR 90–92)	27,5 d (IQR 21-57.5)	**0.002** ^**a**^
First day fortification	9 d (IQR 6–13)	12 d (IQR 10–18)	7 d (IQR 6–11)	**0.028** ^**a**^
Nutritional intakes				
- Liquids (ml/kg/d)	160 (IQR 149–168)	147 (IQR 145–150)	164.5 (IQR 157.5–168)	0.095^a^
- Calories (kcal/kg/d)	130,8 (IQR 125.6-140.4)	139.7 (IQR 122.5-139.9)	130.7 (IQR 126.3-142.2)	0.734^a^
- Proteins (g/kg/d)	3.7 (IQR 3.5–4.5)	3.6 (IQR 3.6–4.3)	3.8 (IQR 3.4–4.5)	0.709^a^
- Calcium (mg/kg/d)	146 (IQR 134.3-183.3)	146 (IQR 141.9-176.5)	150.5 (IQR 134.1-185.5)	0.892^a^
- Phosphorus (mg/kg/d)	83.6 (IQR 72.4-100.4)	87 (IQR 76-93.8)	82.3 (IQR 71.7-100.6)	0.812^a^
- Vitamin D (UI/d)	600 (IQR 600–700)	800 (IQR 500–835)	600 (IQR 600–600)	0.173^a^

BPD: Bronchopulmonary dysplasia; KMC: Kangaroo mother care; PN: Parenteral Nutrition; NGT: Nasogastric Tube. IQR denotes the interquartile range, which represents the range between the 25th and 75th percentiles

a: Mann-Whitney U test; b: Fisher’s exact test

Various pathologies showed a statistical association with MBDP, including bronchopulmonary dysplasia, pneumonia, hypothyroidism, anemia, patent ductus arteriosus, and seizures.

Delayed initiation of Kangaroo mother care was significantly associated with MBDP. MBDP patients had a median delay of 34 days (IQR 8–52) in starting Kangaroo mother care, while non affected patients had a median delay of 4.5 days (IQR 2-11.2) (*p* < 0.008).

MBDP patients had a significantly longer duration of parenteral nutrition, with a median of 15 days compared to 8 days in non-MBDP patients (*p* < 0.007). They also experienced a lower weight gain during the first month of life (17.9 g/day vs. 30.4 g/day, *p* < 0.035) and received exclusive parenteral nutrition for a longer period.

The type of enteral diet, whether fortified human milk or specific formula, did not show a significant association with MBDP. However, a delayed start of human milk fortification was related to MBDP. MBDP patients had a median delay of 12 days (IQR 10–18) in initiating human milk fortification, while non affected patients had a median delay of 7 days (IQR 6–11) (*p* < 0.028).

No statistically significant associations were found between MBDP and daily intake of liquids, calories, proteins, calcium, phosphorus, and vitamin D. Additionally, there was no association between the start day of vitamin supplementation and MBDP.

### Biochemical factors associated with MBDP

At 3–4 weeks of life, MBDP showed significant associations with lower levels of serum phosphorous and FGF23. MBDP patients had lower phosphate and FGF23 levels than non-MBDP patients. They also had higher uCa/uCr ratio levels. Although serum ALP and B-ALP were higher in the MBDP group, the differences were not statistically significant (Table 3). PTH levels were similar between both groups, and none of the three patients with PTH levels above 88 pg/mL were diagnosed with MBDP. After adjusting for gestational age and sex, the odds ratios for phosphate (0.064; 95% CI 0.005-0.8, p 0.033) and FGF23 (0.885; 95% CI 0.79–0.99, p 0.037) remained significant. However, the association weakened when all biochemical mineral metabolism variables were included.

**Table 3:** Biochemical factors associated with MBDP

	All (25)	MBDP (5)	No MBDP (20)	*p*
Phosphate (mg/dL)	6.9 (IQR 6.2–7.3)	5.2 (IQR 4.05–6.35)	7.1 (IQR 6.6–7.37)	**0.003**
Calcium (mg/dL)	9.6 (IQR 9.450-10)	9.6 (IQR 9.25–10.05)	9.65 (IQR 9.42–9.97)	0.918
ALP (IU/L)	333 (IQR 286.5–485)	601 (IQR 291.5-831.5)	323.5 (IQR 276.75–437.5)	0.118
B-ALP (IU/L)	139 (IQR 106–186)	222 (IQR 113–277)	138 (IQR 104–163)	0.213
Creatinine (mg/dL)	0.34 (IQR 0.27–0.38)	0.38 (IQR 0.23–0.615)	0.34 (IQR 0.28–0.38)	0.495
C. Cystatin (mg/L)	1.43 (IQR 1.31–1.56)	1.4 (IQR 1.17–1.81)	1.44 (IQR 1.3–1.57)	0.709
FGF23 (ng/L)	45.9 (IQR 13.02–76.77)	5 (IQR 5-9.8)	53.4 (IQR 42-84.3)	**0.001**
PTH (pg/mL)	58 (IQR 32.25–75.25)	43 (IQR 22.5–66)	61 (IQR 39–78)	0.155
25-OH-Vit D (ng/mL)	27 (IQR 20.5–33)	33 (IQR 27.5–42)	25 (IQR 20-32.75)	0.083
1-25-2OH-Vit D (pg/mL)	102 (IQR 81–141)	97 (IQR 48.5-176.5)	106.5 (IQR 87.75-135.75)	0.941
uP/UCr (mg/mg)	1. 78 (IQR 0.51–2.59)	0.9 (IQR 0.37–1.62)	1,91 (IQR 0.62–2.72)	0.185
TRP (%)	92 (IQR 86.77–97.69)	92.34 (IQR 81.5–98.9)	91.29 (IQR 86.63–97.15)	0.786
uCa/uCr (mg/mg)	0.6 (IQR 0.31–0.79)	0.8 (IQR 0.59–1.44)	0.57 (IQR 0.21–0.75)	**0.049**

*ALP: alkaline phosphatase; B-ALP: bone alkaline phosphatase; FGF23: Fibroblast growth factor-23; PTH: parathormone;* 25-OH-Vit D: *25-hydroxycholecalciferol;* 1-25-2OH-Vit D: 1,25-dihydroxycholecalciferol; uP: urinary phosphorus; uCr: urinary creatinine; TRP: Tubular Reabsorption of Phosphate; uCa: urinary calcium; IQR denotes the interquartile range, which represents the range between the 25th and 75th percentiles

**Fig. 2:**
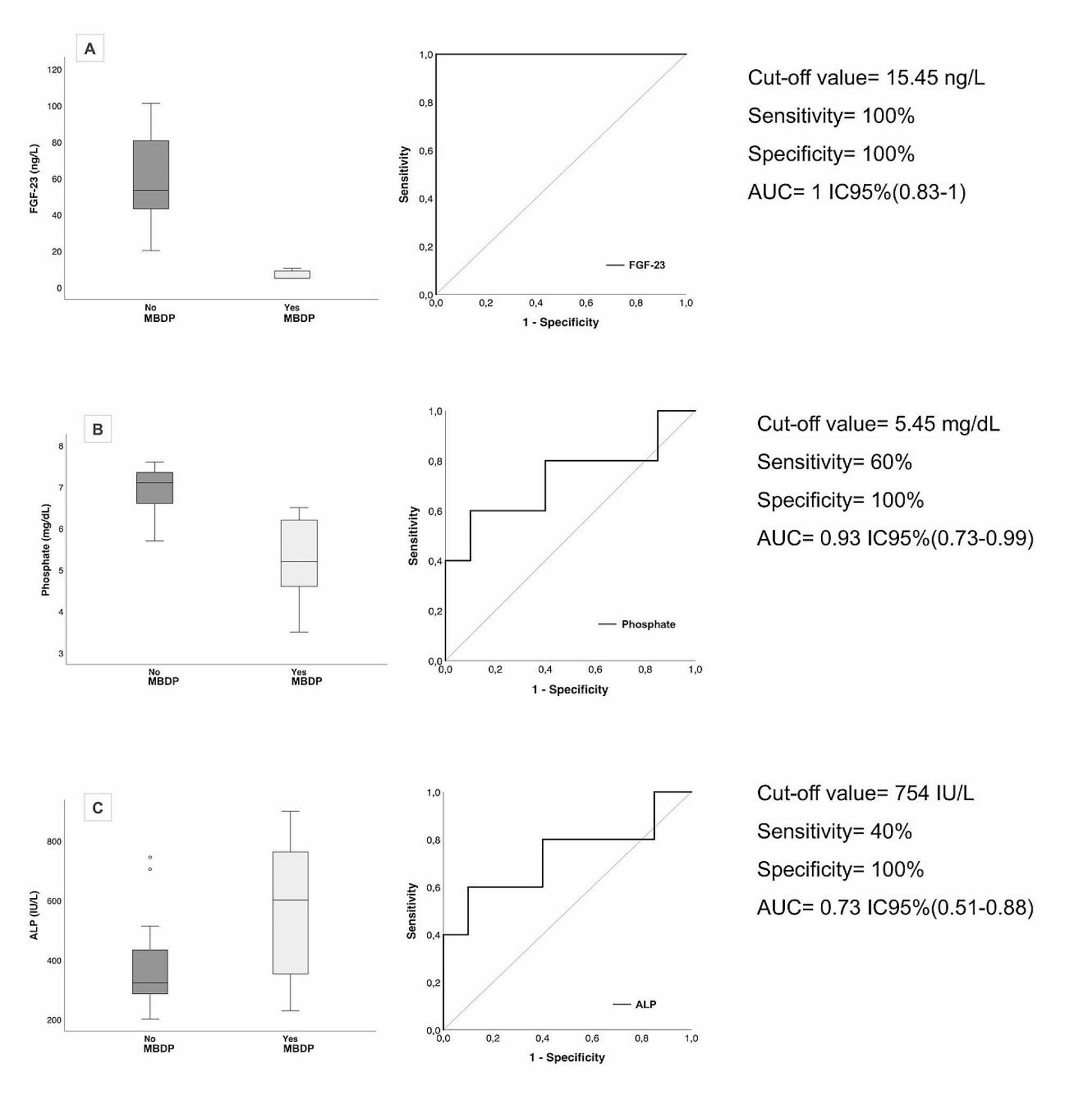
**A**- Box plot demonstrating the distribution of **FGF23** according to metabolic bone disease of prematurity. ROC curve analysis of FGF23 for predicting metabolic bone disease of prematurity. **B-** Box plot demonstrating the distribution of **phosphate** according to metabolic bone disease of prematurity. ROC curve analysis of phosphate for predicting metabolic bone disease of prematurity. **C-** Box plot demonstrating the distribution of **ALP** according to metabolic bone disease of prematurity. ROC curve analysis of ALP for predicting metabolic bone disease of prematurity FGF23: Fibroblast growth factor 23; ALP: alkaline phosphatase; MBDP: Metabolic bone disease of prematurity. AUC: Area under the curve

A box plot with the distribution of FGF23 according to MBDP demonstrates that FGF23 is more effective than conventional parameters (ALP and phosphorus) in identifying the risk for MBDP at 3–4 weeks of life (Fig. 2). The optimal cutoff point for FGF23 in predicting MBDP was determined to be 15.45 ng/L, with a sensitivity and specificity of 100% (95% CI 56.55–100 and 79.61–100, respectively). A phosphorus cutoff point of 4 mg/dl yields a specificity of 100% but a sensitivity of only 20%. However, with a cutoff point of 5.6 mg/dl, the sensitivity increased to 60% while maintaining specificity. For ALP values, a cutoff point of 500 IU/L results in a sensitivity of 60% and specificity of 90%, while a cutoff point of 750 IU/L achieves 100% specificity but a reduced sensitivity of 20%.

In our cohort, only 3 out of 5 (60%) MBDP patients were initially identified as high risk using standard biochemical parameters. However, subsequent follow-up assessments led to a change in classification for the remaining two patients from low risk to high risk. By utilizing FGF23 levels with a cutoff point of 15.45 ng/L, all 5 patients (100%) could have been correctly classified as high risk from 3 to 4 weeks of life, enabling earlier detection and potential adjustments in treatment.

## Discussion

In this study, involving 25 at-risk premature newborns, 20% of them were diagnosed with MBDP. Three of these patients (60%) were identified as high-risk based on biochemical evaluation at 3–4 weeks of age, while the other two patients (40%) were diagnosed in subsequent weeks. Measurement of FGF23 levels at 3–4 weeks of age allows for early identification of high-risk patients and optimization of treatment before other markers become altered. Screening MBDP should be based on prevailing risk factors. According to the guidelines from the American Academy of Pediatrics (AAP), screening for MBDP typically commences around 4–6 weeks after birth, as clinical rickets is rare in the initial weeks of life [16]. Additionally, screening may be warranted if there are indications such as radiographic evidence of rickets or fractures [44, 45].

In our clinical practice setting, biochemical evaluation is typically performed in at-risk patients at 3–4 weeks of age. However, none of the commonly used markers of bone metabolism, such as calcium, phosphorus, ALP, PTH, and vitamin D, are considered specific for MBDP [8, 19, 46].

ALP levels physiologically increase during the first 3 weeks of life, peaking at 6–12 weeks [30]. ALP is expressed in multiple tissues (liver, bone, kidney, intestine, brain, etc.), with over 80% derived from bone and liver, making it a good marker of bone turnover [8]. ALP has been widely used as a marker for MBDP, although there is no agreed cutoff value, and it is not specific, as it can be elevated in other common pathologies, such as cholestasis [47]. ALP levels > 500 U/L have been associated with alterations in bone metabolism, and levels > 700 U/L have been associated with bone demineralization [30, 31]. Viswanathan et al. demonstrated that ALP levels > 500 IU/L in preterm newborns < 30 weeks of gestation are associated with MBDP [48], and Backstrom et al. showed that ALP levels > 900 IU/L in preterm newborns < 33 weeks of gestational age, combined with serum phosphate levels < 5.6 mg/dL, have a diagnostic sensitivity and specificity of 70% and 100%, respectively [29]. In the present study, ALP levels at 3–4 weeks were higher in the MBDP group (601 vs. 323.5 IU/L) but did not reach statistical significance (*p* = 0.118). Even when analyzing the specific bone ALP isoenzyme to exclude confounding factors such as cholestasis, no statistically significant differences were found (222 vs. 138 U/L, *p* = 0.213). Although neonatal practices have evolved significantly over the years, potentially impacting the relevance of the findings to contemporary medical contexts, the use of B-ALP levels did not improve diagnostic performance, as previously shown in the study by Backstrom et al. [29]. Our study demonstrates that although ALP and B-ALP may be markers of MBDP, they are not statistically significant in distinguishing affected individuals in this cohort.

Elevated PTH levels are often seen in preterm infants with MBDP. High PTH levels can indicate secondary hyperparathyroidism, which occurs in response to hypocalcemia and/or hypophosphatemia commonly seen in MBDP. However, there is no general agreement on the PTH reference range for preterm infants. Adult ULN (upper limits of normal) ranges from 50 to 88 pg/ml [32, 49], and elevated PTH levels suggest an increased risk of MBDP [7, 32]. We decided to use the ULN as the cut-off point, although other authors establish even higher cut-off points (100 pg/ml) [7]. In our study, PTH levels were similar between MBDP and non-MBDP patient groups, and none of the three patients with PTH levels above 88 pg/mL were diagnosed with MBDP.

Comparing PTH and FGF23 in preterm infants reveals insights into their roles in mineral metabolism and potential implications for metabolic bone health [50, 51]. PTH regulates calcium and phosphate homeostasis by stimulating bone resorption and renal tubular reabsorption of calcium while promoting renal phosphate excretion. Conversely, FGF23 primarily modulates phosphate levels by inhibiting renal phosphate reabsorption and reducing phosphate absorption from the gut [52]. Although both hormones exhibit alterations in preterm infants, their relationship remains complex and not fully understood, with studies showing variable correlations influenced by factors such as gestational age, birth weight, nutritional status, and comorbidities [53–55].

In terms of urine studies, assessing urinary phosphate and calcium excretion can provide additional insights into mineral metabolism and renal function in preterm infants [56, 57]. A high uCa/uCr ratio was related to low PTH levels, but we did not find a significant association between FGF23 and the uP/uCr ratio or TRP. Changes in urinary phosphate and calcium levels may reflect alterations in renal handling of these minerals, which can be influenced by factors such as gestational age, postnatal age, nutritional intake, and renal maturation. However, in our study there was no correlation between tubular urinary function (uCystatinC/uCr and uGGT/uCr) and gestational age or corrected age.

Further investigations are needed to identify more specific and sensitive markers for the early detection of MBDP in preterm infants. FGF23, a phosphaturic hormone involved in mineral metabolism regulation, has shown promise as a potential marker. In our study, FGF23 levels at 3–4 weeks were significantly lower in the MBDP group than in the control group (5 [5-9.8 ng/L] vs. 53.4 [42-84.3 ng/L], *p* = 0.001). FGF23 levels > 15.45 ng/L had a sensitivity and specificity of 100% (95% CI 56.55–100 and 79.61–100, respectively) for identifying patients with MBDP. These findings suggest that FGF23 measurement may serve as an early diagnostic tool for MBDP in preterm infants.

While our study provides valuable insights, several limitations warrant acknowledgment. Firstly, the relatively small sample size may restrict the generalizability of our findings to broader populations of premature infants. Challenges and limitations exist in measuring FGF23 in preterm infants [58, 59], primarily due to their immature renal function, impacting hormone clearance and metabolism. Variations in sample collection and assay methods may also affect the accuracy and reliability of FGF23 measurements. Moreover, the absence of a control group and longitudinal follow-up data limits our ability to establish causal relationships or determine FGF23’s predictive value for metabolic bone disorders. Additionally, relying on a single time point for FGF23 measurement may overlook dynamic changes in FGF23 levels over time, potentially missing early markers of bone mineralization disorders.

In conclusion, MBDP is a common complication in premature newborns, and early detection is crucial for optimal management. Currently available biochemical markers such as ALP and B-ALP may not be sufficiently specific for MBDP diagnosis. However, FGF23 levels at 3–4 weeks of age show promise as a potential early marker for identifying high-risk patients. Further research is needed to validate these findings and explore additional markers that can improve the diagnostic accuracy and early intervention in MBDP.

### Electronic supplementary material

Below is the link to the electronic supplementary material.

Supplementary Material 1

## Data Availability

The datasets used and/or analyzed during the current study available from the corresponding author on reasonable request.
