# Supplementary material for: Role of fibroblast growth factor-23 as an early marker of metabolic bone disease of prematurity
Source: BMC Pediatr. 2024 Jun 29;24:418. doi: 10.1186/s12887-024-04897-7 (PMC11218264; doi:10.1186/s12887-024-04897-7)
Supplement: Supplementary file 1 — Supplementary Material 1 [file 12887_2024_4897_MOESM1_ESM.docx]

**APENDIX 1. Table.** Nutritional intakes of the study population.

|  | Type of Nutrition | Liquids (ml/kg/d) | Calories (kcal/kg/d) | Protein (g/kg/d) | Calcium (mg/kg/d) | Phosphorus (mg/kg/d) | Vitamin D (IU/d) |
| --- | --- | --- | --- | --- | --- | --- | --- |
| Patient 1 | EN (FHM) | 160 | 131 | 3.89 | 155.7 | 81.07 | 600 |
| Patient 2 | EN (FHM) | 150 | 122.5 | 3.65 | 146 | 76 | 870 |
| Patient 3 | EN (PF) | 138 | 100.7 | 2.77 | 110.77 | 66.46 | 400 |
| Patient 4 | EN (PF) | 167 | 121.9 | 3.3 | 133.3 | 80 | 600 |
| Patient 5 | EN (FHM + MCTs) | 144 | 140.4 | 4.79 | 201 | 108.2 | 800 |
| Patient 6 | EN (FHM + MCTs) | 169 | 139.7 | 3.63 | 141.9 | 72.4 | 800 |
| Patient 7 | EN (FHM + MCTs) | 147 | 146 | 4.28 | 176.5 | 93.8 | 800 |
| Patient 8 | EN (FHM) | 160 | 144 | 4.96 | 206.4 | 110.4 | 800 |
| Patient 9 | EN (FHM + MCTs) | 149 | 140.1 | 3.92 | 159.1 | 83.6 | 600 |
| Patient 10 | EN (FHM + MCTs) | 160 | 137.7 | 3.36 | 130.4 | 66.4 | 600 |
| Patient 11 | EN (FHM) | 145 | 130.6 | 4.51 | 187.6 | 100.36 | 600 |
| Patient 12 | EN (FHM) | 168 | 130.3 | 3.54 | 137.3 | 69.9 | 800 |
| Patient 13 | EN (HM) + PN | 150 | 108 | 3,65 | 28 | 87 | 400 |
| Patient 14 | EN (FHM + MCTs) | 167 | 160.5 | 4.75 | 195.1 | 103.42 | 600 |
| Patient 15 | EN (AC) | 155 | 125.6 | 4.49 | 179.8 | 119.35 | 600 |
| Patient 16 | EN (FHM) | 170 | 130.5 | 3.47 | 133.8 | 67.8 | 600 |
| Patient 17 | EN (FHM) | 164 | 128.8 | 3.59 | 140.3 | 71.89 | 600 |
| Patient 18 | EN (PF) | 174 | 127 | 3.48 | 139.3 | 83.6 | 600 |
| Patient 19 | EN (PF) | 168 | 122.6 | 3.36 | 134.4 | 80.64 | 600 |
| Patient 20 | EN (FHM) | 174 | 147.9 | 4.7 | 191.3 | 100.87 | 600 |
| Patient 21 | EN (FHM+ PF ) | 160 | 120.4 | 3.28 | 129.2 | 71.6 | 600 |
| Patient 22 | EN (FHM + MCTs) | 171 | 149.6 | 4.17 | 166.86 | 86.86 | 600 |
| Patient 23 | EN (FHM + MCTs) | 167 | 153.3 | 4.5 | 183.3 | 96.7 | 600 |
| Patient 24 | EN (FHM + MCTs) | 145 | 139.9 | 4.51 | 187.64 | 100.36 | 600 |
| Patient 25 | EN (FHM) | 165 | 130.8 | 3.69 | 145.4 | 74.82 | 600 |

EN: Enteral Nutrition, PN: Parenteral Nutrition, FHM: Fortified Human Milk, PF: Preterm Formula 16%, AC: Alprem Clinic®, MCTs: Medium Chain Triglycerides.
